# Tryptophan-Dependent Control of Colony Formation After DNA Damage via Sea3-Regulated TORC1 Signaling in *Saccharomyces cerevisiae*

**DOI:** 10.1534/g3.115.018721

**Published:** 2015-05-04

**Authors:** Erica J. Polleys, Alison A. Bertuch

**Affiliations:** * Integrative Molecular and Biomedical Sciences Graduate Program, Baylor College of Medicine, Houston, Texas 77030; † Department of Pediatrics and Department of Molecular & Human Genetics, Baylor College of Medicine, Houston, Texas 77030

**Keywords:** Sea3, Iml1 complex, TORC1, tryptophan, DNA damage

## Abstract

The *Saccharomyces cerevisiae* Iml1 complex inhibits TORC1 signaling and SEACAT antagonizes the Iml1 complex. Conditions in which SEACAT functions to inhibit Iml1 and, hence, TORC1 signaling, remain largely unknown. The SEACAT member Sea3 was linked previously to telomere maintenance and DNA repair via genome-wide genetic and physical interaction studies. Therefore, we questioned whether Sea3 functioned through TORC1 to influence these pathways. Deletion of *SEA3* delayed the emergence of telomerase-independent survivors that use break-induced replication (BIR) to maintain their telomeres. Similarly, *sea3∆* mutants exhibited a delay in colony formation in a BIR assay strain after double-strand break (DSB) induction as well as on the DNA-damaging agent bleomycin. Deletion of *IML1* rescued the impaired growth of *sea3∆* mutants after DNA damage, consistent with Sea3 functioning as a regulator of TORC1 signaling. The delay was not attributable to slowed DSB repair or termination of the DNA damage checkpoint but to tryptophan auxotrophy. High levels of tryptophan in yeast peptone dextrose media did not rescue the delay in colony formation, suggesting a defect in tryptophan import, although levels of the high-affinity tryptophan permease Tat2 were not perturbed in the *sea3*Δ mutant. Addition of quinolinic acid, an intermediate of the *de novo* NAD+ biosynthetic pathway, however, rescued the delay in colony formation in the *sea3*Δ mutant. Together, these findings highlight the importance of enforcement of TORC1 signaling and suggest that internal tryptophan levels influence growth recovery post DNA damage through the role of tryptophan in NAD+ synthesis.

Sea3 is a member of the vacuolar SEA complex, a dynamic complex of four proteins [Iml1 (Sea1), Sea2–4], which associates with the nucleoporins Seh1 and Sec13 as well as the TORC1 regulators Npr2 and Npr3 (Dokudovskaya *et al.* 2011). Initial studies in budding yeast implicated the SEA complex in the response to nitrogen starvation, amino acid biogenesis, and intracellular trafficking (Dokudovskaya *et al.* 2011), processes shared with the Tor complex TORC1 (Takahara and Maeda 2013). Given this and its interaction with TORC1 regulators, it seemed likely that the SEA complex functioned in TORC1 signaling. Consistent with this, human homologs of the SEA complex components were identified as upstream regulators of mTORC1, distributing into two regulatory complexes, GATOR1 and GATOR2 (Bar-Peled *et al.* 2013). The GATOR1 complex contains the Iml1 homolog, DEPDC5, as well as Nprl2 and Nprl3, whereas the GATOR2 complex contains the Sea2, Sea3, and Sea4 homologs, WDR24, WDR59, and Mios, respectively, as well as the homologs to yeast Seh1 and Sec13. This initial study revealed that GATOR2 functions as a positive regulator of mTORC1 by negatively regulating GATOR1, which is an inhibitor of mTORC1 signaling.

Further analysis of the SEA complex in *S. cerevisiae* revealed two epistatic groups, the Iml1 complex (*a.k.a*. SEACIT) and SEACAT, which segregate and function like GATOR1 and GATOR2 in humans; SEACAT (Sea2, Sea3, Sea4, Seh1, and Sec13) negatively regulates the Iml1 complex/SEACIT (Iml1, Npr2, and Npr3), which negatively regulates TORC1 signaling (Figure 1A) (Panchaud *et al.* 2013a,b). Importantly, although there are two Tor complexes in *S. cerevisiae*, TORC1 and TORC2, each of which regulate a distinct set of cellular functions, (Loewith *et al.* 2002), the SEA complex has only been established as a regulator of TORC1. Analysis of TORC1 signaling under conditions of amino acid deprivation revealed that Sea2, Sea3, and Sea4 act redundantly to attenuate the inhibitory properties of the Iml1 complex (Panchaud *et al.* 2013a). Whether there are conditions under which an individual member of the SEACAT complex functions alone to regulate TORC1 signaling remains unknown.

**Figure 1:**
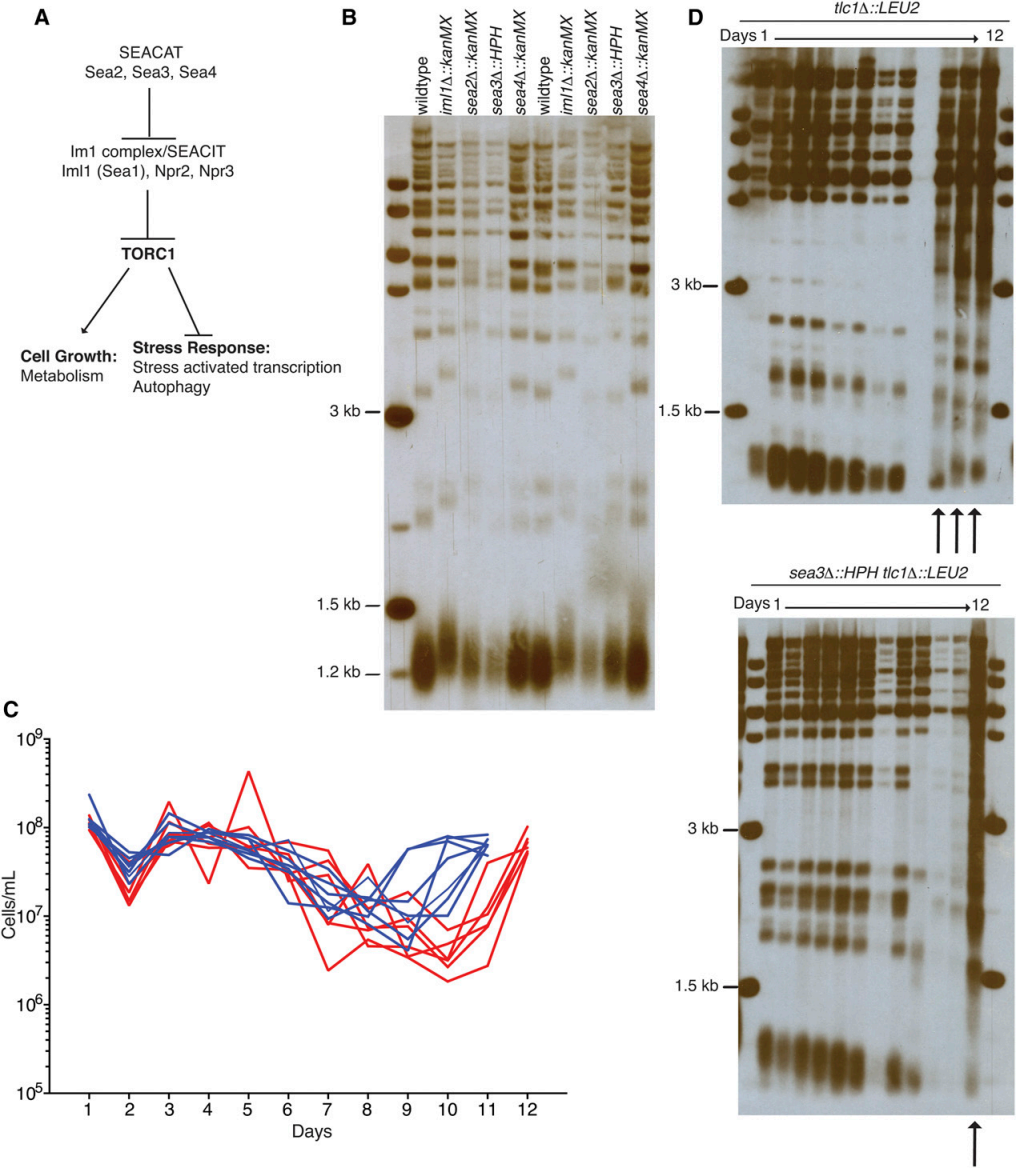
Sea3 impacts senescence progression and the formation of survivors in the absence of telomerase. (A) Proposed pathway indicating that SEACAT, which contains Sea2, Sea3, and Sea4, acts as a negative regulator of Iml1 complex/SEACIT, which contains Iml1, Npr2, and Npr3. The Iml1 complex/SEACIT functions as a negative regulator of TORC1 signaling, which regulates a variety of downstream targets. (B) Southern blot of *Xho*I-digested genomic DNA isolated from wild-type and haploid *SEA* complex gene deletion mutants were probed with a telomere repeat specific probe. Approximately two thirds of wild-type telomeres, which contain Y´ elements in the subtelomeric region, give rise to a 1.2-kb terminal restriction fragment upon *Xho*I digestion. The restriction fragments of greater length derive, in part, from individual telomeres that lack a Y´ element. (C) Liquid culture senescence progression assays of *tlc1∆* and *sea3∆ tlc1∆* haploids obtained from sporulation and microdissection of a *sea3*Δ/*SEA3 tlc1*Δ/*TLC1* diploid. Blue lines indicate growth curves of individual *tlc1*Δ spores (n = 7), and red lines indicate growth curves of individual *sea3*Δ *tlc1*Δ spores (n = 5). (D) As in (B) except genomic DNA was isolated daily from samples in the liquid senescence progression assay (C). Arrows indicate the emergence of Type II survivors, which acquired a more heterogeneous banding pattern.

Inhibition of TORC1 signaling induces a variety of cellular changes indicative of a starvation response, including a reduction in protein synthesis, enlargement of the vacuole, activation and repression of gene transcription, and induction of autophagy (Zoncu *et al.* 2011). Amino acid biosynthesis and sorting of amino acid permeases are also impacted when TORC1 is inhibited in response to starvation. For example, various high affinity amino acid permeases are relocalized in the cell, changing the import of certain classes of amino acids. Well-established examples of this are the effects on the high-affinity tryptophan permease, Tat2, and the general amino acid permease, Gap1 (Beck *et al.* 1999). In rich media, Tat2 is stable and imports tryptophan. Upon the inhibition of TORC1 signaling that results from nitrogen deprivation, the phosphatase Tap42 dephosphorylates Npr1, a serine/threonine kinase, rendering Npr1 active. Activated Npr1 then mediates the degradation of Tat2 and localization of Gap1 to the plasma membrane (Schmidt *et al.* 1998; Beck *et al.* 1999). Consequently, Gap1 becomes responsible for the import of amino acids, including tryptophan. Notably, several members of the SEA complex have genetic interactions with factors that regulate Gap1 localization, such as Lst8, a component of the TOR signaling pathway, and share fitness profiles across numerous chemical and environmental stress conditions with genes involved in Gap1 sorting (Dokudovskaya *et al.* 2011; Hillenmeyer *et al.* 2008).

In addition to the aforementioned downstream targets, TORC1 signaling in budding yeast also influences telomere length maintenance and the repair of DNA double-stranded breaks (DSBs) via nonhomologous end joining. This is achieved, in part through the control of the levels of Ku (Ungar *et al.* 2011), a heterodimeric protein that has functions at both telomeric ends and DNA ends created by DSBs (Boulton and Jackson 1996). Interestingly, *SEA3* was identified in a screen for gene deletions that altered the growth of a strain bearing *cdc13-1*, a temperature-sensitive allele of the telomeric binding protein Cdc13 (Addinall *et al.* 2008). Genes that when deleted resulted in a synthetically sick phenotype at the permissive temperature when combined with the *cdc13-1* mutation were given the maintenance of telomeric capping (MTC) designation, whereas genes that when deleted synthetically rescued the *cdc13-1* lethality at the nonpermissive temperature were given the rescue of telomeric capping (RTC) designation. Members of both MTC and RTC designations were varied in their known or putative biological functions. In this initial study, *SEA3* was placed in the MTC group and, as a previously anonymous gene, given the name *MTC5*. A subsequent study, however, reported *mtc5∆* (*sea3∆*) synthetically rescued the *cdc13-1* lethality at the nonpermissive temperature, thus exhibiting an RTC phenotype (Addinall *et al.* 2011). Given this discrepancy and the results that follow, we have used the *SEA3* rather than *MTC5* gene designation in this report.

TORC1 also contributes to the DNA damage response (DDR) secondary to DSBs. Treatment of cells with rapamycin, which directly inhibits the TORC1 complex, and then with the DNA-damaging agent methyl methanesulfonate results in suppression of the Rad53-dependent up-regulation of ribonucleotide reductase (*RNR*) genes, *RNR1* and *RNR3*, and decreased survival (Shen *et al.* 2007). In addition, strains bearing *RNR* gene deletions have decreased survival when treated with both rapamycin and methyl methanesulfonate, leading to the hypothesis that survival of cells incurring DNA damage requires TORC1 enforcement of nucleotide pools sufficient for DNA synthesis postdamage. Interestingly, Sea3 has been found in genome-wide screens to physically interact with Yku80, Srs2, and Rfa3, all of which are DNA repair proteins (Ho *et al.* 2002; Chiolo *et al.* 2005).

Given the role of TORC1 signaling in telomere maintenance and the DDR, and the potential connection of the upstream TORC1 regulator Sea3 with telomere and DNA repair proteins, we sought to determine whether Sea3 played a regulatory role in these processes. We found that deletion of *SEA3* did not impact telomere length in telomerase-positive strains but did alter the progression of senescence in the absence of telomerase and delayed the emergence of telomerase-independent survivors that maintain their telomeres via break-induced replication (BIR). Similarly, *sea3*Δ mutants had a delay in colony formation after DSB induction in a strain that assays for BIR and upon exposure to the DNA-damaging agent bleomycin. We found Sea3 to function, as predicted, upstream of TORC1 through Iml1 after DSB induction. The studies unveil a novel type of recovery defect, which is not in the termination of the DDR, but rather in intracellular tryptophan. They implicate tight regulation of TORC1 signaling via Sea3 in the ability of the cell to achieve sufficient intracellular tryptophan to allow timely recovery after DNA damage.

## Materials and Methods

### Strains and plasmids

The strains and plasmids used in this paper are described in Supporting Information, Table S1. Deletion and epitope-tagged strains were generated by one-step gene replacement or integration, respectively, with the noted selectable marker. All incubations were performed at 28°.

### Telomere analysis

Telomere length analysis and determination of telomerase-independent survivors were performed as described previously (Lendvay *et al.* 1996).

### Senescence progression assays

Serial liquid culture senescence progression assays were carried out as described previously (Le *et al.* 1999; Rizki and Lundblad 2001). To summarize, spore colonies from freshly dissected tetrads were inoculated in their entirety into YPD media and grown at 28° for 46 hr. Cell counts were determined via hemocytometer. Samples were diluted back into fresh media to a concentration of 1 × 10^5^ cells/mL and grown for 22 hr at which time cells were counted. The cultures were again diluted back into fresh media to a concentration of 1 × 10^5^ cells/mL. Cell counts and dilution into fresh media were repeated every 22 hr. Several isolates of each genotype were examined.

### Growth experiments

To assess growth in the BIR assay strain, fivefold serial dilutions of exponentially growing liquid cultures in YPLactate were plated on YPD or YPGal. To assay strains containing plasmids, the cultures were pre-grown in -Leu, -Trp, or -Ura minimal media, as appropriate, to maintain selection of the plasmid. To assess growth under conditions of stress induction, cultures were pregrown in either YPD for the YPH strain or YPLactate for the BIR assay strain and plated for single colonies on YPD medium containing 2−4 µg/mL bleomycin, 0.25% glucose, 0.5 M NaCl, or 3 mM hydrogen peroxide or at 37°. To assess growth under conditions of added tryptophan, fivefold serial dilutions of liquid cultures grown in YPLactate were plated on YPD, YPD + 100 μM tryptophan, YPGal, and YPGal + 100 μM tryptophan. To assess growth with added quinolinic acid, fivefold serial dilutions of liquid cultures grown in YPLactate were plated on YPD, YPD + 2 μM or 4 μM quinolinic acid, YPGal, and YPGal + 2 μM or 4 μM quinolinic acid. All phenotypes were recorded 4 days postplating unless otherwise specified.

### BIR assays

BIR plating and determination of percent viability were performed as described previously (Lydeard *et al.* 2007). Sensitivity to canavanine and/or hygromycin determined whether repair occurred via BIR or another pathway. All individual colonies on the YPGal plate were picked and inoculated into 96-well dishes. The cell suspensions were then pinned onto YPD, canavanine and hygromycin plates.

### HO induction and repair kinetics

Strains were pregrown in YPLactate. Galactose induction, sample collection and processing, and DNA analysis were performed as described previously (Lydeard *et al.* 2010).

### Protein analysis under galactose induction

Strains were pregrown in YPLactate to approximately 0.5 × 10^7^ cells. Initial aliquots were taken and then galactose was added to each culture to a final concentration of 2%. Aliquots were taken at the indicated time points. Samples were spun at 3000 rpm and cell pellets washed twice with water before being frozen at −80°. The cell pellets were thawed and normalized to cell count before lysis. For analysis of Rad53 phosphorylation, protein lysates were prepared by trichloroacetic acid method as previously described (Foiani *et al.* 1994). For analysis of Tat2 protein levels, samples were prepared as previously described (Abe and Iida 2003). Before western analysis of Tat2, 50 µg of whole-cell extract was denatured in 5% SDS and 5% β-mercaptoethanol at 37° for 10 m. Western blots were probed with α-Flag (F3165; Sigma-Aldrich), α-Rad53 (provided by M. Foiani), and α-PGK (ab113687; Abcam) antibodies.

## Results

### Sea3 impacts senescence progression and the formation of survivors in the absence of telomerase

We initially were interested in examining the impact of the SEA complex genes on telomere maintenance as both *sea2∆* and *sea3∆* deletions were identified as modifiers of growth of a *cdc13-1* strain and the *sea2∆* mutant was found to have short telomeres when examined in a genome-wide screen (Addinall *et al.* 2008, 2011; Askree *et al.* 2004). We found *sea2∆*, *sea3∆*, and *sea4∆* haploid deletion strains had telomere lengths comparable with wild-type, whereas the *iml1∆* strain had slightly longer telomeres (Figure 1B). Thus, in this directed analysis in the YPH274 strain background, deletion of the SEA complex genes had little, if any impact, on telomere length.

Whereas wild-type strains, which constitutively express telomerase, maintain stable telomere length with propagation, strains deficient in telomerase, such as those lacking the telomerase regulatory subunits, Est1 or Est3, the telomerase catalytic subunit, Est2, or the telomerase RNA subunit, Tlc1, experience progressive telomere shortening, which leads to eventual cellular senescence (Lendvay *et al.* 1996; Lundblad and Szostak 1989; Singer and Gottschling 1994). Interestingly, the *sea3∆* allele was reported to synthetically interact with the *est1∆* deletion in a genome-wide screen for genes that affected telomere-driven senescence progression and recovery (Chang *et al.* 2011). Therefore, we examined directly the effect of a *sea3*Δ mutation on senescence progression in the absence of *TLC1*. To determine this, we investigated telomere length and growth potential of *sea3∆ tlc1∆* strains compared with *tlc1∆* strains derived from *sea3Δ/SEA3tlc1∆/TLC1* (telomerase-proficient) diploids. The *sea3Δ/SEA3tlc1∆/TLC1* diploids were sporulated and dissected, and individual meiotic segregants were inoculated into liquid culture. After 22 hr of growth, the cell concentration was determined and the cultures diluted back daily in a standard liquid culture senescence progression assay (Bertuch and Lundblad 2004; Le *et al.* 1999). Although the *sea3∆* single-mutant strains exhibited a brief and slight decline in growth potential on early days of the experiment, they were otherwise largely indistinguishable from wild-type with respect to growth over the course of the experiment (Figure S1A), consistent with previous reports (Dokudovskaya *et al.* 2011; Panchaud *et al.* 2013a). Additionally, the single *sea3*Δ mutants, like wild-type, did not undergo progressive telomere shortening but maintained a stable telomere length over time (Figure S1B). We did find, however, that, with continued propagation, the *sea3*Δ *tlc1*Δ mutants had a more prolonged duration of restricted growth compared with *tlc1*Δ mutants alone, with a continued decline in cell numbers beyond day 9 and 10, until growth recovery on day 11 or 12 (Figure 1C). This finding was somewhat different than the genome-wide study, in which *sea3*Δ (*mtc5∆) est1*Δ mutants were found to have *rad52Δ est1Δ* mutant characteristics, with accelerated entry into senescence and lack of growth recovery (Chang *et al.* 2011). The differences in the growth patterns may have been attributable to differences in the media [liquid (this study) *vs.* solid (Chang *et al.* 2011)], media components, or strain background [YPH274 (this study) *vs.*, BY4741 (Chang *et al.* 2011)].

Two types of telomerase-independent survivors have been described, both of which rely on BIR to elongate telomeres in the absence of telomerase (Lundblad and Blackburn 1993; Lydeard *et al.* 2007; Teng and Zakian 1999). Type I survivors amplify subtelomeric Y´ elements and have short terminal telomeric repeat tracts, whereas Type II survivors amplify telomeric sequence from the very end of another telomere, resulting in highly heterogeneous lengths of terminal telomeric repeat tracts (Lundblad and Blackburn 1993; Teng and Zakian 1999). Type II survivors predominant in serial liquid culture assays because of a growth advantage over Type I survivors (Teng and Zakian 1999). We found that *sea3∆ tlc1*Δ mutants still formed Type II survivors in liquid culture, but the survivors took approximately 1−2 d longer to be visualized than for *tlc1∆* mutants (Figure 1D), indicating a possible delay in the execution of BIR or in growth post survivor formation. Thus, although the absence of Sea3 did not impact normal telomere length maintenance, it did impact growth and recovery of telomerase-independent survivors.

### Absence of Sea3 slows colony formation after DSB induction

**Figure 2:**
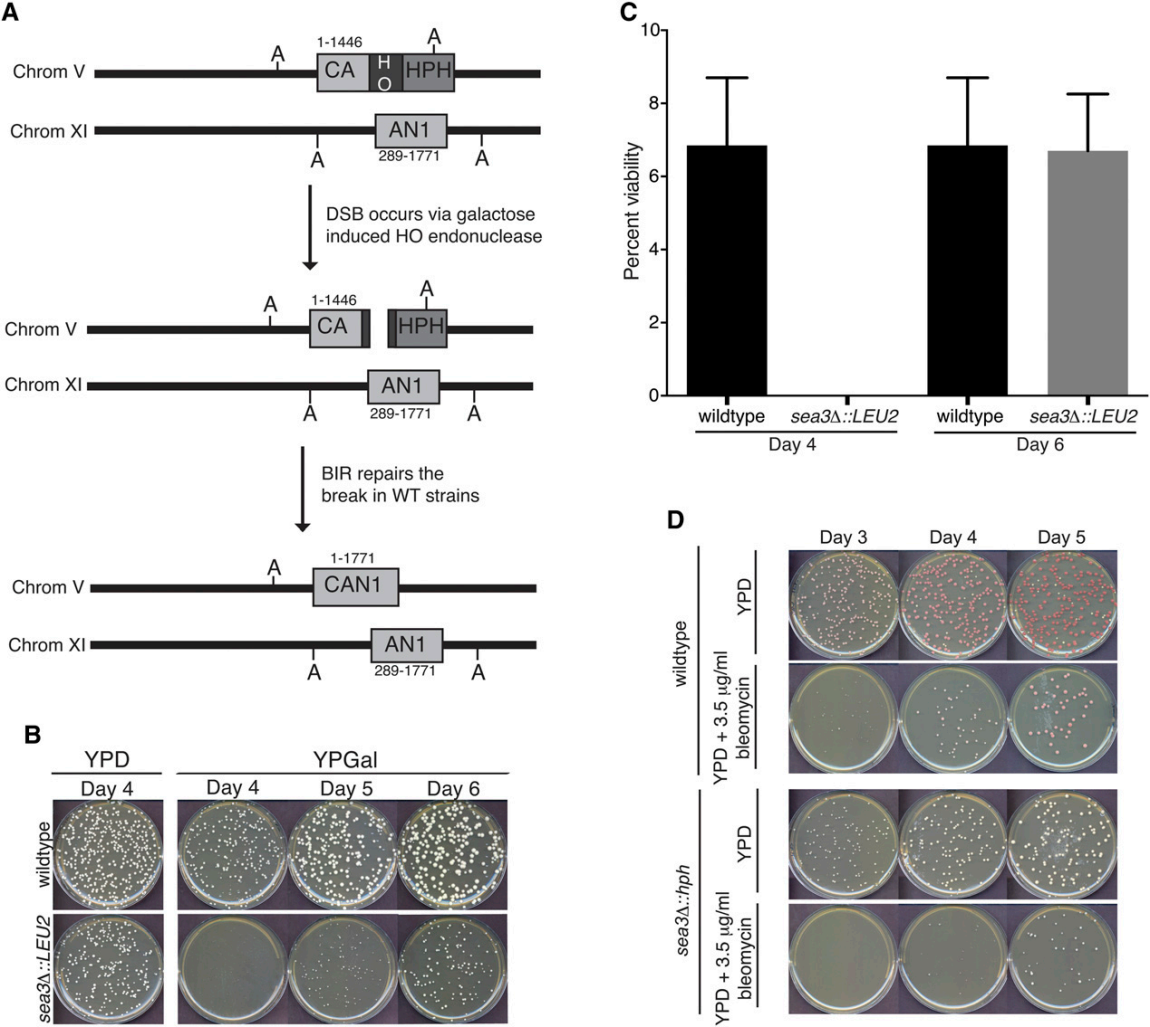
Loss of Sea3 impacts colony formation in the break-induced replication (BIR) assay strain and on bleomycin. (A) BIR assay strain (Lydeard *et al.* 2010). An HO cut site (HO), marked with *HPH*, is integrated into the *CAN1* gene (represented as CA) on chromosome V, deleting the 3′ portion of *CAN1*. The *CAN1* donor (represented as AN1), which shares 1157 bp of homology to the *CAN1* gene, is integrated into chromosome XI. Sites marked with A indicate *Ava*I sites used for monitoring BIR repair in Figure 3B. (B) Platings for single colonies of wild-type and *sea3*Δ mutants in the BIR assay strain on YPD and YPGal. (C) Percent viability of wild-type and *sea3*Δ mutants as a ratio of number of colonies on YPGal divided by the dilution factor and then divided by the number of colonies on YPD. Values represent average of two independent trials and error bars indicate standard error of the mean (SEM). (D) Platings for single colonies of wild-type and *sea3*Δ mutants in the YPH274 genetic background on YPD and YPD + 3.5 μg/mL bleomycin.

As the *sea3∆* mutation slowed the appearance of BIR-dependent survivors in the absence of telomerase, we wanted to determine whether Sea3 specifically impacted BIR. To assess this, we deleted *SEA3* in a strain created previously to analyze BIR at an induced DSB (Lydeard *et al.* 2007). In this strain, *CAN1*, which confers sensitivity to canavanine and is present on the nonessential telomere-proximal end of the recipient chromosome (Chr V), is truncated by the insertion of an HO endonuclease site flanked by a hygromycin resistance (*HPH*) reporter (Figure 2A). In addition, a 5′ truncation of *CAN1* is inserted on the donor chromosome (Chr XI), resulting in 1157 bp of *CAN1* homology between the donor and recipient chromosomes. The HO endonuclease is under the control of a galactose inducible promoter. Upon exposure to galactose, the DSB generated at the HO site on Chr V is repaired via BIR, resulting in a full-length *CAN1* gene and loss of the hygromycin reporter. When we deleted *SEA3* in this strain, we noted a consistent 2-d delay in the formation of quantifiable colonies on galactose compared with wild-type, whereas the *sea3∆* mutants formed colonies comparably with wild-type on glucose (Figure 2B and Figure S2, uninduced). The overall viability on galactose, however, was equivalent to wild-type (Figure 2C). Importantly, the effect on galactose was attributable to loss of the *SEA3* gene product as it was complemented by expression of *SEA3* on a CEN plasmid (Figure S3A).

To determine whether the growth delay was caused by a general sensitivity to galactose (rather than the DSB induced by galactose), we took *sea3*Δ mutants in the BIR strain background that had been plated previously on galactose, had undergone BIR repair of the DSB and, therefore, would not sustain another HO-induced DSB when replated on galactose, and compared their growth on galactose to *sea3*Δ mutants that had not been previously exposed to galactose and, therefore, would undergo DSB induction when plated on galactose. If the growth delay of the *sea3*Δ mutants on galactose were simply due to a general sensitivity to galactose, then the presence or absence of a cleavable HO cut site would not matter and both types of *sea3*Δ mutants would be equally sensitive to galactose. We found, however, that when the *sea3*Δ mutants that were originally plated on galactose were re-plated on galactose, quantifiable colonies appeared sooner (Figure S2; see also Figure 5A and Figure S8A), suggesting that the delay in colony formation in the *sea3*Δ mutant was not simply due to a sensitivity to galactose.

To further explore the growth delay in the absence of Sea3 after DSB induction, we examined the growth of *sea3*Δ mutants in the YPH274 genetic background on the DSB-inducing agent bleomycin. The *sea3∆* mutant strain was sensitive bleomycin and, again, formed colonies more slowly than wild-type (Figure 2D). Growth of the *sea3∆* mutant strain on galactose was comparable with the wild-type (Figure S3B), thus, clearly demonstrating a growth defect attributable to DSBs and independent of galactose. Additionally, like the delay in the *sea3∆* BIR assay strain background when plated on galactose, the delay in colony formation in the *sea3∆* mutant strain on bleomycin was complemented by expression of *SEA3* on a CEN plasmid (Figure S3B). Therefore, absence Sea3 resulted in delayed growth upon DNA damage in two different genetic backgrounds—either via an inducible DSB in the BIR assay strain or by the DNA-damaging agent bleomycin in the YPH274 background.

### *sea3*Δ mutants are not classical recovery mutants

The delay in colony formation in the *sea3∆* mutant BIR assay strain suggested that *sea3****∆*** mutants may have a delay in recovery post-DNA damage. Recovery is typically defined as resumption of mitosis after repair is completed and the checkpoint is turned off; recovery mutants demonstrate sustained activation of the DDR despite repair of the DSB (Vaze *et al.* 2002). A variety of proteins have been implicated in recovery and most are associated with the DNA damage checkpoint or repair (Guillemain *et al.* 2007; Leroy *et al.* 2003; Vaze *et al.* 2002).

**Figure 3:**
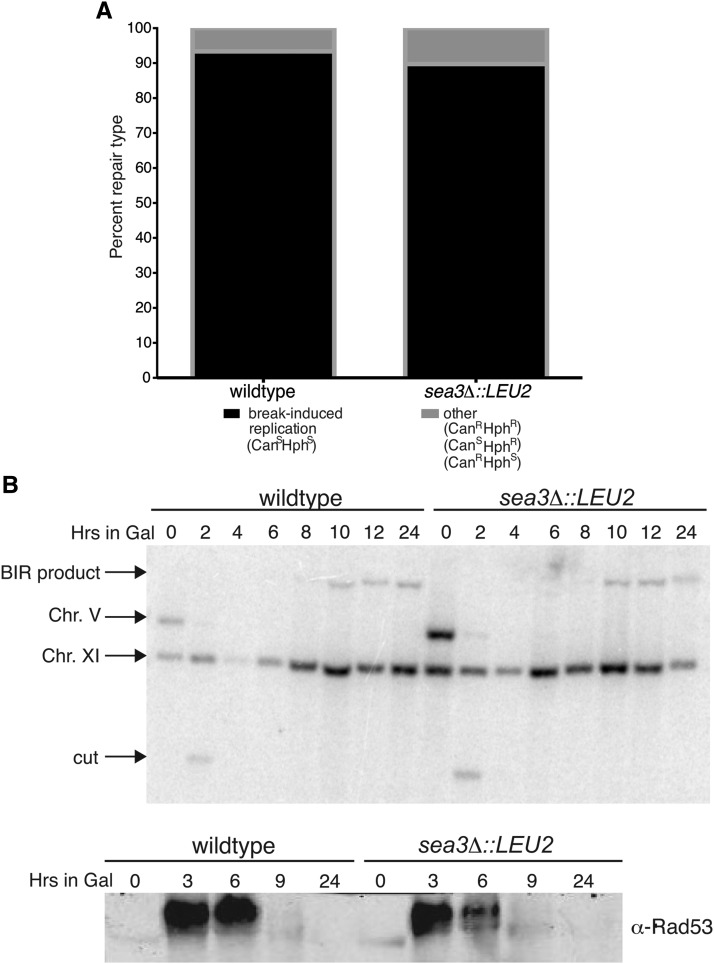
Loss of Sea3 does not delay break-induced replication (BIR) repair or extinction of the DNA damage checkpoint. (A) Percent repair types observed in wild-type and *sea3*Δ BIR assay strain mutants from (Figure 2B), determined by plating on media containing canavanine or hygromycin. Values represent average of two independent trials. (B) *Ava*I-digested genomic DNA collected from wild-type and *sea3*Δ BIR assay strain mutants at designated hours postgalactose induction blotted and probed with the *CAN1* gene. (C) Rad53 western blot analysis of whole-cell extracts of an equivalent number of wild-type and *sea3*Δ mutant cells were prepared via the trichloroacetic acid method at designated hours postgalactose induction. Image is representative of two independent experiments.

We, therefore, first determined whether the *sea3*Δ mutant BIR assay strain was able to repair the HO-induced DSB via BIR. Like wild-type, it repaired via BIR a majority of the time, as tracked by the sensitivity of the colonies to canavanine and hygromycin (Figure 3A). Additionally, the *sea3*Δ mutant repaired the DSB as rapidly as wild-type with repair products appearing in the interval between 8 and 10 hr after DSB induction (Figure 3B). Next we determined whether the mutant had a delay in terminating the DNA damage checkpoint postrepair, which is most frequently monitored by examining the pattern of Rad53 hyperphosphorylation after DSB induction. We found, however, that the *sea3*Δ mutant did not have prolonged Rad53 hyper-phosphorylation as compared to wild-type (Figure 3C). Taken together, as the *sea3*Δ mutant was able to repair the DSBs and extinguish the DNA damage checkpoint as proficiently as the wild-type, the delay in colony formation post DSB induction was distinct from a classical recovery defect.

### Sea3 functions through the TORC1 pathway in response to DNA damage

**Figure 4:**
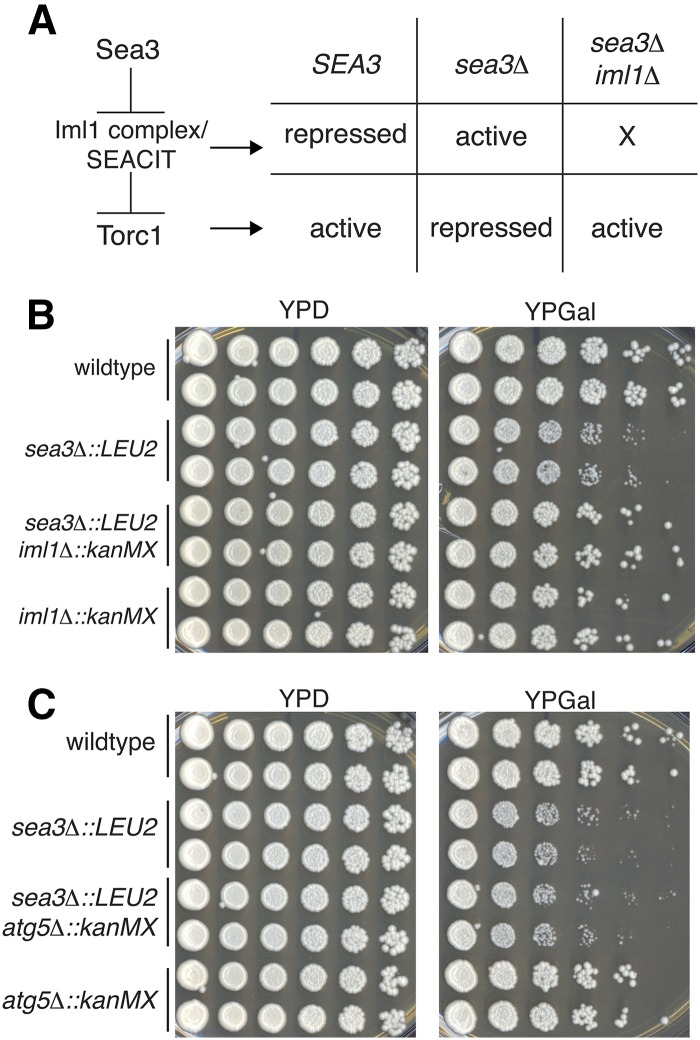
Sea3 functions through TORC1, but not autophagy, in response to DNA damage. (A) Proposed effects on TORC1 if Sea3 functions as a negative regulator of the Iml1 complex/SEACIT. (B) Fivefold serial dilutions of wild-type and *iml1*Δ, *sea3*Δ *iml1*Δ, and *sea3*Δ BIR assay strain mutants plated on YPD and YPGal. (C) Fivefold serial dilutions of wild-type and *atg5*Δ, *sea3*Δ *atg5*Δ, and *sea3*Δ BIR assay strain mutants plated on YPD and YPGal.

Given Sea3′s role as a negative regulator of the Iml1 complex (SEACIT), which in turn negatively regulates TORC1 (Panchaud *et al.* 2013a,b), we reasoned that the delay in colony formation in the *sea3∆* BIR assay strain might be the result of hyperrepression of TORC1 (Figure 4A). If so, then deletion of *IML1* would rescue the delay. This is precisely what we observed (Figure 4B). Similarly, we found that deletion of *IML1* rescued the *sea3*Δ mutant growth delay on bleomycin in the YPH274 strain background (Figure S4). Thus, these data are consistent with Sea3 functioning through TORC1 and this regulation of TORC1 impacting colony formation post-DNA repair in the BIR assay strain.

To determine whether Sea3 and, perhaps, TORC1 signaling were required for growth in response to other stress conditions, we plated *sea3∆* mutants in both the YPH274 and BIR assay strain backgrounds on YPD containing a low concentration of glucose (0.25% compared to 2% in standard YPD), high salt (0.5 M NaCl added to standard YPD), or hydrogen peroxide (3 mM H_2_O_2_ added to standard YPD), and at high temperature (37°). We found the *sea3*Δ mutant in the YPH274 strain background had a growth delay on medium containing high salt, an effect that was mediated through TORC1 signaling as it was rescued by deletion of *IML1* (Figure S5A). However, a growth delay was not observed on high salt with the *sea3∆* mutant in the BIR assay strain background (Figure S5B), suggesting the phenotype was influenced by strain specific factors and not solely the absence of Sea3. In both strain backgrounds, we found that Sea3 was not required for growth in response to any of the other stresses tested (Figure S5). Taken together, although there are some strain specific differences, *sea3*Δ mutants experience a growth defect under conditions that induce DSBs and under conditions of high salt in a TORC1-dependent manner.

We next looked for possible targets downstream of TORC1 that might be responsible for the delay in colony formation phenotype. Likely candidates were factors mediating autophagy, which is negatively regulated by TORC1 signaling, and previously identified to be a pathway downstream of the yeast SEA complex (Dokudovskaya *et al.* 2011; Takahara and Maeda 2013). If Sea3 functioned to promote TORC1 repression of autophagy and, thereby, regulate growth post-DNA repair, then a block in autophagy would rescue the delay observed in the *sea3∆* mutant. However, we found that deletion of *ATG5*, which encodes a core autophagy factor (Mizushima *et al.* 1998), had no impact on the delay in the BIR assay strain (Figure 4C). Thus, the delay in colony formation in the *sea3∆* mutant was not due to aberrant up-regulation of autophagy but rather due to misregulation of another downstream TORC1 target.

### The growth delay in *sea3*Δ mutants is rescued by the presence of wild-type *TRP1*

**Figure 5:**
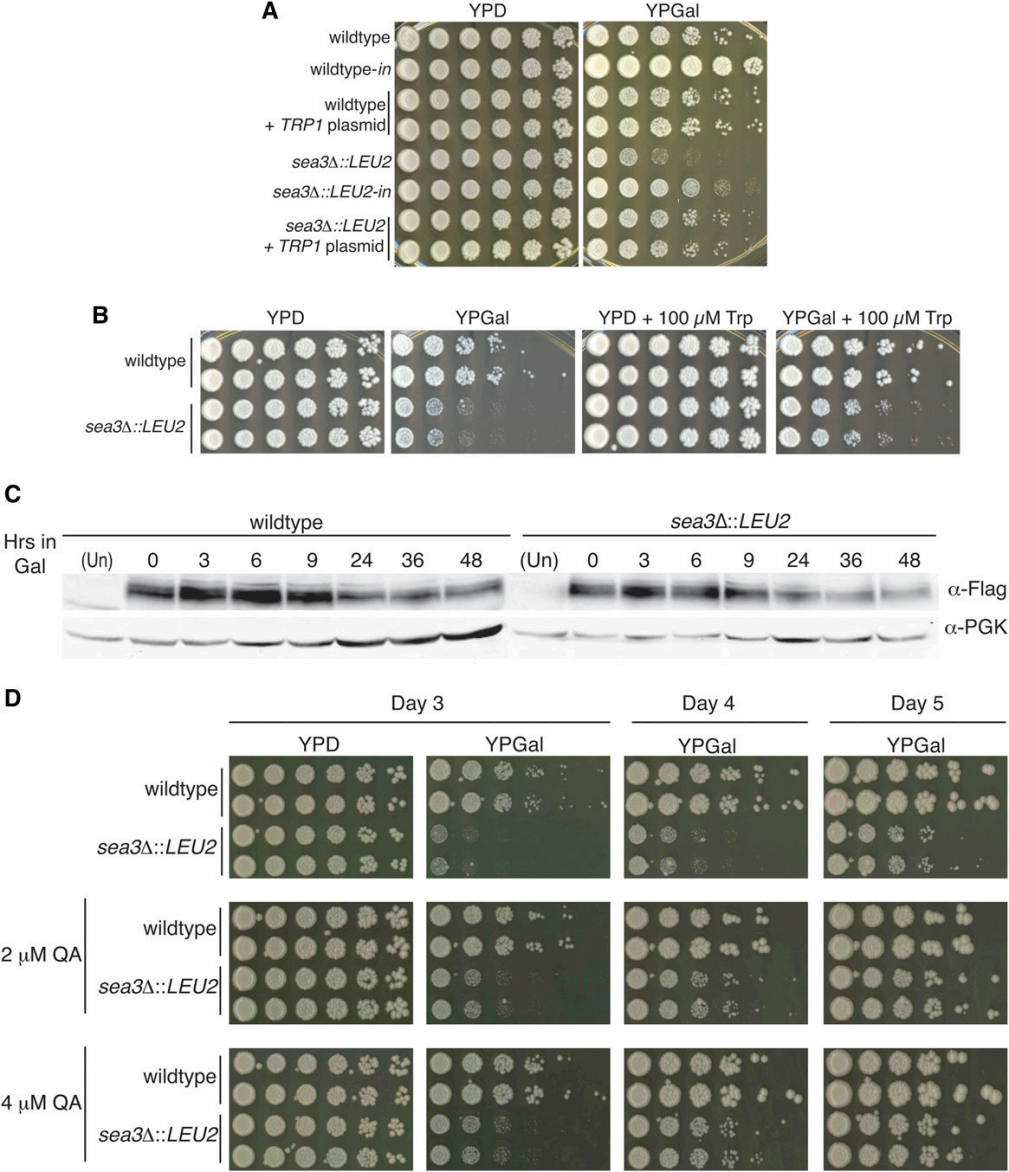
Expression of *TRP1*, but not exogenous tryptophan, rescues the delay in colony formation in *sea3Δ* mutants. (A) Fivefold serial dilutions of wild-type and *sea3*Δ mutants in the break-induced replication (BIR) assay strain with and without the addition of the *TRP1* plasmid pRS414 on YPD and YPGal. The wild-type-*in* and *sea3∆*::*LEU2-in* strains have been previously experienced HO-induction with galactose and undergone a repair event, rendering them unable to undergo HO-mediated DSB induction upon replating on galactose. (B) Fivefold serial dilutions of the wild-type and *sea3*Δ BIR assay strain mutants plated on YPD, YPGal, YPD + 100 μM tryptophan (Trp) and YPGal + 100 μM Trp. (C) Western blots showing Tat2-3XFlag levels post-galactose induction at the indicated time points. Whole-cell extracts were prepared and blotted with α-Flag. Images are representative of three independent experiments. (D) Fivefold serial dilutions of the wild-type and *sea3*Δ mutants in the BIR assay strain were plated on YPD, YPGal and YPD and YPGal with either 2 or 4 μM quinolinic acid (QA) added. Plates were imaged on day 3, day 4, and day 5.

Both the SEA complex and TORC1 signaling have been linked to amino acid biosynthesis and internal trafficking of amino acid permeases. In the case of the SEA complex, the link has been inferred from genome-wide pairwise fitness screen data (Beck *et al.* 1999; Dokudovskaya *et al.* 2011; Schmidt *et al.* 1998). Therefore, it was possible that the delay in colony formation after DNA damage was attributable to a change in amino acid requirements. The parental BIR assay strain is auxotrophic for the amino acids tryptophan and leucine as well as the nucleobase uracil due to *trp1*, *leu2*, and *ura3* mutations, respectively. Therefore, we asked whether addition of wild-type copies of these genes impacted the growth of wild-type and *sea3*Δ mutant strains. Introduction of a *LEU2* CEN plasmid into the parental wild-type BIR assay strain did not result in a growth delay upon plating on galactose (Figure S6A), whereas deletion of *SEA3* with a *kanMX* cassette did (Figure S6B), indicating that the growth delay of the *sea3*Δ::*LEU2* mutant BIR strain on galactose was not due to *LEU2* expression. Surprisingly, however, introduction of a *TRP1* CEN plasmid rescued the delay of the *sea3∆* mutant (Figure 5A). The rescue was specific to *TRP1*, as introduction of a *URA3* CEN plasmid did not similarly rescue the delay (Figure S3A). Likewise, *TRP1* rescued the growth delay observed when the *sea3∆*::*HPH* mutant in the YPH274 (*trp1*-) background was plated on bleomycin, although to a partial extent (Figure S7). These results suggested that the delay in colony formation observed in *sea3∆* mutants was due to perturbations in internal levels of tryptophan.

Therefore, we examined whether tryptophan availability was contributing to the *sea3∆* mutant phenotype. Even in rich media, the level of tryptophan is low (measured 26 µg/mL in Jarolim *et al.* 2013), so we reasoned that if tryptophan availability were the source of the delay in colony formation in the *sea3*Δ mutant, addition of an excessive amount of tryptophan to the media might rescue the delay. Although we found that both wild-type and *sea3∆* mutant strains grew slightly more robustly on YPGal supplemented with an additional 100 μM tryptophan compared with YPGal, the *sea3*Δ mutant still displayed a growth delay relative to the wild-type (Figure 5B). Combined with the rescue of *TRP1* expression, this suggested that after DNA damage, *sea3*Δ mutants are either unable to import tryptophan into the cell sufficiently or that tryptophan consumption was altered.

To address this further, we examined whether the levels of Tat2 changed in the absence of Sea3 upon galactose induction in the BIR assay strain. We found that Tat2 levels were comparable throughout the time course in wild-type and the *sea3*Δ mutant, with levels declining between 32 and 48 hr (Figure 5C and Figure S9). Additionally, deletion of *TAT2* in the BIR assay strain did not result in a delay in colony formation post-DSB induction (Figure S10), suggesting that although the delay in colony formation in the *sea3*Δ mutant appeared to be attributable to insufficient internal tryptophan levels, loss of Tat2 protein was not responsible.

Of note, we also found that the delay in colony formation in the *sea3∆* BIR assay strain was only observed on rich media as, when the *sea3∆* mutant was plated on synthetic defined media containing galactose and tryptophan, among the added amino acids (*e.g.*, -Ura Gal or -Leu Gal minimal media; tryptophan concentration 40 µg/mL), it grew like the wild-type (Figure S6 and Figure S8). This indicated that, under those nutrient conditions, the cells were sufficient in tryptophan uptake.

One possibility for the delay in colony formation in the *sea3*Δ mutant might relate to the availability of the coenzyme nicotinamide adenosine dinucleotide (NAD+), which is synthesized either via a salvage pathway or *de novo* from tryptophan via the kynurenine pathway. Notably, NAD+ is used in a wide range of cellular pathways, including those involved in DNA repair and the DDR (Kato and Lin 2014). We hypothesized that the slow growth phenotype of the *sea3*Δ mutant post-DNA damage could be due to limiting NAD+ levels resulting from decreased internal tryptophan. Therefore, supplying an intermediate downstream from tryptophan in the *de novo* synthesis pathway might rescue the delay. Quinolinic acid is one such intermediate and, importantly, can be imported into cells via the high affinity nicotinic acid permease and, thereby, used to increase internal levels of NAD+ (Ohashi *et al.* 2013). When we plated wild-type and *sea3*Δ mutants in the BIR strain background on media containing galactose and varying amounts of quinolinic acid, we found the *sea3*Δ mutants grew markedly better than without quinolinic acid (Figure 5D). Therefore, we conclude tryptophan auxotrophy is synthetic with the *sea3∆* mutation under DNA damage conditions partially due to a decline in the levels of the coenzyme NAD+.

## Discussion

In this study, we have shown that Sea3 is critical for the recovery of growth post-DNA damage caused by DSBs and in the absence of telomerase. We also have shown that the growth delay observed in *sea3∆* mutants after DNA damage is downstream of TORC1 signaling. Though a link between TORC1 signaling and the adaptation to unrepaired DSBs has been reported, this has been shown to be via TORC1 inhibition of autophagy (Dotiwala *et al.* 2013). Similarly, inhibition of autophagy by TORC1 signaling has been implicated in DNA damage sensitivity (Dyavaiah *et al.* 2011; Robert *et al.* 2011). This study provides evidence that TORC1 signaling has a role in recovery post-DNA damage independent of autophagy, a role related to the availability of tryptophan, and, consequently, the *de novo* pathway of NAD+ synthesis, and enforced by Sea3.

Sea3 was placed previously in the SEACAT epistatic group because of its functional redundancy with Sea2 and Sea4 in response to nutrient conditions (Panchaud *et al.* 2013b). Therefore, although, it was previously established that inhibition of TORC1 signaling results in shortened telomeres via alteration of the levels of Ku (Ungar *et al.* 2011), the normal telomere length we observed in haploids bearing *a SEA2*, *SEA3* or *SEA4* gene deletion (Figure 1B) may have been due to Sea2, Sea3, and Sea4 functioning redundantly to inhibit the Iml1 complex and support TORC1 signaling as they do in response to nutrients (Panchaud *et al.* 2013b).

We also, however, identified a role for Sea3 that could not be compensated for by the continued presence of Sea2 and Sea4, which is in the recovery following DNA damage, including at telomeres (Figure 1, Figure 2, and Figure S3). Additional evidence supports the possibility that members of the SEACAT epistasis group have disparate functions. For example, although Sea2 and Sea3 are structurally similar and both genetically interact with *cdc13-1* (Addinall *et al.* 2008, 2011), Sea3 has an RWD domain, structurally similar to an E2 ubiquitin conjugating enzyme, which is not present in Sea2 (Dokudovskaya *et al.* 2011). Additionally, *sea2*Δ homozygous mutants have a sporulation defect not observed with deletion of other SEA complex member genes [data not shown and (Briza *et al.* 2002)]. Together, these results suggest that individual components of the SEACAT epistasis group may function in different circumstances to regulate TORC1 signaling.

The finding that the delay in colony formation in *sea3*Δ mutants after DNA damage was not caused by defects in the ability or kinetics of repair nor prolonged activation of the DNA damage checkpoint (Figure 3) was surprising because no studies have shown a growth defect post-DNA repair without accompanying Rad53 hyperphosphorylation. Instead, we found the delay was dependent on tryptophan auxotrophy (Figure 5A), uncovering the importance of tryptophan after DNA damage. Consistent with this finding, Tat2 protein levels increased in both wild-type and the *sea3*Δ mutant postbreak induction when Rad53 hyperphosphorylation was high but declined more slowly, suggesting that, even as the DNA damage checkpoint was alleviated, the demand for tryptophan import remained elevated (Figure 3C, Figure 5C, and Figure S9).

The tryptophan auxotrophy-dependent delay in colony formation in the *sea3∆* BIR assay strain on rich, but not synthetic, media (Figure S8) is reminiscent of the growth phenotype of yeast with mutations in *ELM1*, which encodes a serine/threonine kinase involved in cell growth and division (Garrett 1997). In previous studies, *elm1* mutant strains were found to have a different set of phenotypes depending on whether they were auxotrophic or prototrophic for tryptophan. In addition, the *elm1* mutant phenotype was observed on rich media but not on synthetic defined media containing tryptophan. Examination of the activity of several amino acid permeases revealed Gap1 activity was inappropriately decreased in cells deficient in both Elm1 and internal tryptophan. Thus, rather than Tat2, misregulation of Gap1, a known target of TORC1 signaling, may be responsible for the delay in colony formation upon DNA damage in the *sea3∆* mutant. Indeed, a delay in colony formation upon DNA damage was not observed in the *tat2∆* strain (Figure S10), indicating an alternate pathway for tryptophan import upon DNA damage.

In addition to being an essential substrate for protein synthesis, tryptophan is required for the *de novo* synthesis of NAD+. We found the delay in colony formation in the *sea3*Δ mutant was partially suppressed by the addition of quinolinic acid (Figure 5D), an intermediate downstream from tryptophan in the NAD+ *de novo* synthesis pathway, suggesting an increased demand for NAD+ postDNA damage. NAD+ has a variety of roles in both cellular growth and DNA transactions; however, the specific activities driving such an increased demand remain unknown. An increase demand for NAD+ synthesis has been reported in the context of DNA damage signaling emanating from the telomere in the *cdc13-1* mutant at the semipermissive temperature, reflected by marked up-regulation of expression of *BNA2*, which, like tryptophan, is required for *de novo* NAD+ synthesis. Notably, this was independent of the NAD+-dependent deacetylase Sir2, which has known functions at the telomere (Greenall *et al.* 2008). *TRP1* also was identified in a genome-wide screen as a suppressor of the *cdc13-1* phenotype at the restrictive temperature (Addinall *et al.* 2011). As the addition of quinolinic acid only partially suppressed the delay in colony formation in the *sea3*Δ mutant, additional NAD+-independent pathways could be perturbed post-DNA damage. For example, an increased demand for tryptophan might also be due to an increase in protein synthesis during the DDR. Further studies are needed to determine the NAD+-dependent and -independent pathways underlying the increased demand for tryptophan post-DNA damage.

In summary, these findings reveal a new class of DNA recovery mutants, distinct from those defective in terminating the DDR, that impact the status of tryptophan, an important determinant of growth post-DNA damage. Several of the commonly used yeast strains, including S288c, W303, SEY6210, YPH274, and their derivatives, are auxotrophic for tryptophan as the result of *trp1* mutations. *TRP1* is also a commonly used selectable and counterselectable marker. Thus, this work raises the possibility that the DNA damage sensitivity phenotype of some previously identified genes may be a synthetic phenotype with a background *trp1* mutation. Conversely, mutations rescuing a DNA damage phenotype could be caused by the use of *TRP1* as the selectable marker. Addressing whether a DNA damage phenotype manifests if a strain is auxotrophic for tryptophan or other nutrients may provide important insight into the potential pathway it affects in response to DNA damage.

The SEACAT and the Iml1 complex are functionally conserved in humans. To date, somatic mutations in the human *IML1*/*SEA1* homolog, *DEPDC5*, have been found mutated in cancers (Bar-Peled *et al.* 2013) and germline mutations have been found in familial autosomal-dominant epilepsy and focal malformations of cortical development (Dibbens *et al.* 2013; Ishida *et al.* 2013; Martin *et al.* 2013; Picard *et al.* 2014; D’Gama *et al.* 2015). It is anticipated that homologs of other members of the SEA complex, including *SEA3*, will also prove to be clinically relevant.

## References

[bib1] Abe F. Iida H., 2003 Pressure-induced differential regulation of the two tryptophan permeases Tat1 and Tat2 by ubiquitin ligase Rsp5 and its binding proteins, Bul1 and Bul2. Mol. Cell. Biol. 23: 7566–7584. 14560004 10.1128/MCB.23.21.7566-7584.2003 PMC207609

[bib2] Addinall S. G. Downey M. Yu M. Zubko M. K. Dewar J., 2008 A genomewide suppressor and enhancer analysis of cdc13–1 reveals varied cellular processes influencing telomere capping in *Saccharomyces cerevisiae*. Genetics 180: 2251–2266. 18845848 10.1534/genetics.108.092577 PMC2600956

[bib3] Addinall S. G. Holstein E. M. Lawless C. Yu M. Chapman K., 2011 Quantitative fitness analysis shows that NMD proteins and many other protein complexes suppress or enhance distinct telomere cap defects. PLoS Genet. 7: e1001362. 21490951 10.1371/journal.pgen.1001362 PMC3072368

[bib4] Askree S. H. Yehuda T. Smolikov S. Gurevich R. Hawk J., 2004 A genome-wide screen for *Saccharomyces cerevisiae* deletion mutants that affect telomere length. Proc. Natl. Acad. Sci. USA 101: 8658–8663. 15161972 10.1073/pnas.0401263101 PMC423251

[bib5] Bar-Peled L. Chantranupong L. Cherniack A. D. Chen W. W. Ottina K. A., 2013 A tumor suppressor complex with GAP activity for the Rag GTPases that signal amino acid sufficiency to mTORC1. Science 340: 1100–1106. 23723238 10.1126/science.1232044 PMC3728654

[bib6] Beck T. Schmidt A. Hall M. N., 1999 Starvation induces vacuolar targeting and degradation of the tryptophan permease in yeast. J. Cell Biol. 146: 1227–1238. 10491387 10.1083/jcb.146.6.1227 PMC2156124

[bib7] Bertuch A. A. Lundblad V., 2004 EXO1 contributes to telomere maintenance in both telomerase-proficient and telomerase-deficient *Saccharomyces cerevisiae*. Genetics 166: 1651–1659. 15126387 10.1534/genetics.166.4.1651 PMC1470828

[bib8] Boulton S. J. Jackson S. P., 1996 Identification of a *Saccharomyces cerevisiae* Ku80 homologue: roles in DNA double strand break rejoining and in telomeric maintenance. Nucleic Acids Res. 24: 4639–4648. 8972848 10.1093/nar/24.23.4639 PMC146307

[bib9] Briza P. Bogengruber E. Thur A. Rutzler M. Munsterkotter M., 2002 Systematic analysis of sporulation phenotypes in 624 non-lethal homozygous deletion strains of *Saccharomyces cerevisiae*. Yeast 19: 403–422. 11921089 10.1002/yea.843

[bib10] Chang H.Y. Lawless C. Addinall S.G. Oexle S. Taschuk M., 2011 Genome-wide analysis to identify pathways affecting telomere-initiated senescence in budding yeast. G3 (Bethesda) 1: 197–208. 22384331 10.1534/g3.111.000216 PMC3276134

[bib11] Chiolo I. Carotenuto W. Maffioletti G. Petrini J. H. Foiani M., 2005 Srs2 and Sgs1 DNA helicases associate with Mre11 in different subcomplexes following checkpoint activation and CDK1-mediated Srs2 phosphorylation. Mol. Cell. Biol. 25: 5738–5751. 15964827 10.1128/MCB.25.13.5738-5751.2005 PMC1156977

[bib12] D’Gama A. M. Geng Y. Couto J. A. Martin B. Boyle E. A., 2015 Mammalian target of rapamycin pathway mutations cause hemimegalencephaly and focal cortical dysplasia. Ann. Neurol. 77: 720–725. 25599672 10.1002/ana.24357 PMC4471336

[bib13] Dibbens L. M. de Vries B. Donatello S. Heron S. E. Hodgson B. L., 2013 Mutations in DEPDC5 cause familial focal epilepsy with variable foci. Nat. Genet. 45: 546–551. 23542697 10.1038/ng.2599

[bib14] Dokudovskaya S. Waharte F. Schlessinger A. Pieper U. Devos D.P., 2011 A conserved coatomer-related complex containing Sec13 and Seh1 dynamically associates with the vacuole in *Saccharomyces cerevisiae*. Mol Cell Proteomics 10: M110.006478. 21454883 10.1074/mcp.M110.006478 PMC3108837

[bib15] Dotiwala F. Eapen V. V. Harrison J. C. Arbel-Eden A. Ranade V., 2013 DNA damage checkpoint triggers autophagy to regulate the initiation of anaphase. Proc. Natl. Acad. Sci. USA 110: E41–E49. 23169651 10.1073/pnas.1218065109 PMC3538254

[bib16] Dyavaiah M. Rooney J. P. Chittur S. V. Lin Q. Begley T. J., 2011 Autophagy-dependent regulation of the DNA damage response protein ribonucleotide reductase 1. Mol. Cancer Res. 9: 462–475. 21343333 10.1158/1541-7786.MCR-10-0473

[bib17] Foiani M. Marini F. Gamba D. Lucchini G. Plevani P., 1994 The B subunit of the DNA polymerase alpha-primase complex in *Saccharomyces cerevisiae* executes an essential function at the initial stage of DNA replication. Mol. Cell. Biol. 14: 923–933. 8289832 10.1128/mcb.14.2.923-933.1994 PMC358447

[bib18] Garrett J. M., 1997 The control of morphogenesis in Saccharomyces cerevisiae by Elm1 kinase is responsive to RAS/cAMP pathway activity and tryptophan availability. Mol. Microbiol. 26: 809–820. 9427410 10.1046/j.1365-2958.1997.6231990.x

[bib19] Greenall A. Lei G. Swan D. C. James K. Wang L., 2008 A genome wide analysis of the response to uncapped telomeres in budding yeast reveals a novel role for the NAD+ biosynthetic gene BNA2 in chromosome end protection. Genome Biol. 9: R146. 18828915 10.1186/gb-2008-9-10-r146 PMC2760873

[bib20] Guillemain G. Ma E. Mauger S. Miron S. Thai R., 2007 Mechanisms of checkpoint kinase Rad53 inactivation after a double-strand break in *Saccharomyces cerevisiae*. Mol. Cell. Biol. 27: 3378–3389. 17325030 10.1128/MCB.00863-06 PMC1899965

[bib21] Hillenmeyer M. E. Fung E. Wildenhain J. Pierce S. E. Hoon S., 2008 The chemical genomic portrait of yeast: uncovering a phenotype for all genes. Science 320: 362–365. 18420932 10.1126/science.1150021 PMC2794835

[bib22] Ho Y. Gruhler A. Heilbut A. Bader G. D. Moore L., 2002 Systematic identification of protein complexes in Saccharomyces cerevisiae by mass spectrometry. Nature 415: 180–183. 11805837 10.1038/415180a

[bib23] Ishida S. Picard F. Rudolf G. Noe E. Achaz G., 2013 Mutations of DEPDC5 cause autosomal dominant focal epilepsies. Nat. Genet. 45: 552–555. 23542701 10.1038/ng.2601 PMC5010101

[bib24] Jarolim S. Ayer A. Pillay B. Gee A.C. Phrakaysone A., 2013 *Saccharomyces cerevisiae* genes involved in survival of heat shock. G3 (Bethesda) 3: 2321–2333. 24142923 10.1534/g3.113.007971 PMC3852394

[bib25] Kato M. Lin S. J., 2014 Regulation of NAD+ metabolism, signaling and compartmentalization in the yeast *Saccharomyces cerevisiae*. DNA Repair (Amst.) 23: 49–58. 25096760 10.1016/j.dnarep.2014.07.009 PMC4254062

[bib26] Le S. Moore J. K. Haber J. E. Greider C. W., 1999 RAD50 and RAD51 define two pathways that collaborate to maintain telomeres in the absence of telomerase. Genetics 152: 143–152. 10224249 10.1093/genetics/152.1.143 PMC1460580

[bib27] Lendvay T. S. Morris D. K. Sah J. Balasubramanian B. Lundblad V., 1996 Senescence mutants of *Saccharomyces cerevisiae* with a defect in telomere replication identify three additional EST genes. Genetics 144: 1399–1412. 8978029 10.1093/genetics/144.4.1399 PMC1207693

[bib28] Leroy C. Lee S. E. Vaze M. B. Ochsenbein F. Guerois R., 2003 PP2C phosphatases Ptc2 and Ptc3 are required for DNA checkpoint inactivation after a double-strand break. Mol. Cell 11: 827–835. 12667463 10.1016/s1097-2765(03)00058-3

[bib29] Loewith R. Jacinto E. Wullschleger S. Lorberg A. Crespo J. L., 2002 Two TOR complexes, only one of which is rapamycin sensitive, have distinct roles in cell growth control. Mol. Cell 10: 457–468. 12408816 10.1016/s1097-2765(02)00636-6

[bib30] Lundblad V. Szostak J. W., 1989 A mutant with a defect in telomere elongation leads to senescence in yeast. Cell 57: 633–643. 2655926 10.1016/0092-8674(89)90132-3

[bib31] Lundblad V. Blackburn E. H., 1993 An alternative pathway for yeast telomere maintenance rescues est1- senescence. Cell 73: 347–360. 8477448 10.1016/0092-8674(93)90234-h

[bib32] Lydeard J. R. Jain S. Yamaguchi M. Haber J. E., 2007 Break-induced replication and telomerase-independent telomere maintenance require Pol32. Nature 448: 820–823. 17671506 10.1038/nature06047

[bib33] Lydeard J. R. Lipkin-Moore Z. Jain S. Eapen V. V. Haber J. E., 2010 Sgs1 and exo1 redundantly inhibit break-induced replication and de novo telomere addition at broken chromosome ends. PLoS Genet. 6: e1000973. 20523895 10.1371/journal.pgen.1000973 PMC2877739

[bib34] Martin C. Meloche C. Rioux M. F. Nguyen D. K. Carmant L., 2014 A recurrent mutation in DEPDC5 predisposes to focal epilepsies in the French-Canadian population. Clin. Genet. 86: 570–574. 24283814 10.1111/cge.12311

[bib35] Mizushima N. Noda T. Yoshimori T. Tanaka Y. Ishii T., 1998 A protein conjugation system essential for autophagy. Nature 395: 395–398. 9759731 10.1038/26506

[bib36] Ohashi K. Kawai S. Murata K., 2013 Secretion of quinolinic acid, an intermediate in the kynurenine pathway, for utilization in NAD+ biosynthesis in the yeast *Saccharomyces cerevisiae*. Eukaryot. Cell 12: 648–653. 23457190 10.1128/EC.00339-12 PMC3647768

[bib37] Panchaud N. Peli-Gulli M. P. De Virgilio C., 2013 a Amino acid deprivation inhibits TORC1 through a GTPase-activating protein complex for the Rag family GTPase Gtr1. Sci. Signal. 6: ra42. 23716719 10.1126/scisignal.2004112

[bib38] Panchaud N. Peli-Gulli M. P. De Virgilio C., 2013 b SEACing the GAP that nEGOCiates TORC1 activation: evolutionary conservation of Rag GTPase regulation. Cell Cycle 12: 2948–2952. 23974112 10.4161/cc.26000 PMC3875668

[bib39] Picard F. Makrythanasis P. Navarro V. Ishida S. de Bellescize J., 2014 DEPDC5 mutations in families presenting as autosomal dominant nocturnal frontal lobe epilepsy. Neurology 82: 2101–2106. 24814846 10.1212/WNL.0000000000000488

[bib40] Rizki A. Lundblad V., 2001 Defects in mismatch repair promote telomerase-independent proliferation. Nature 411: 713–716. 11395777 10.1038/35079641

[bib41] Robert T. Vanoli F. Chiolo I. Shubassi G. Bernstein K. A., 2011 HDACs link the DNA damage response, processing of double-strand breaks and autophagy. Nature 471: 74–79. 21368826 10.1038/nature09803 PMC3935290

[bib42] Schmidt A. Beck T. Koller A. Kunz J. Hall M. N., 1998 The TOR nutrient signalling pathway phosphorylates NPR1 and inhibits turnover of the tryptophan permease. EMBO J. 17: 6924–6931. 9843498 10.1093/emboj/17.23.6924 PMC1171040

[bib43] Shen C. Lancaster C. S. Shi B. Guo H. Thimmaiah P., 2007 TOR signaling is a determinant of cell survival in response to DNA damage. Mol. Cell. Biol. 27: 7007–7017. 17698581 10.1128/MCB.00290-07 PMC2168917

[bib44] Singer M. S. Gottschling D. E., 1994 TLC1: template RNA component of *Saccharomyces cerevisiae* telomerase. Science 266: 404–409. 7545955 10.1126/science.7545955

[bib45] Takahara T. Maeda T., 2013 Evolutionarily conserved regulation of TOR signalling. J. Biochem. 154: 1–10. 23698095 10.1093/jb/mvt047

[bib46] Teng S. C. Zakian V. A., 1999 Telomere-telomere recombination is an efficient bypass pathway for telomere maintenance in *Saccharomyces cerevisiae*. Mol. Cell. Biol. 19: 8083–8093. 10567534 10.1128/mcb.19.12.8083 PMC84893

[bib47] Ungar L. Harari Y. Toren A. Kupiec M., 2011 Tor complex 1 controls telomere length by affecting the level of Ku. Curr. Biol. 21: 2115–2120. 22169538 10.1016/j.cub.2011.11.024

[bib48] Vaze M. B. Pellicioli A. Lee S. E. Ira G. Liberi G., 2002 Recovery from checkpoint-mediated arrest after repair of a double-strand break requires Srs2 helicase. Mol. Cell 10: 373–385. 12191482 10.1016/s1097-2765(02)00593-2

[bib49] Zoncu R. Efeyan A. Sabatini D. M., 2011 mTOR: from growth signal integration to cancer, diabetes and ageing. Nat. Rev. Mol. Cell Biol. 12: 21–35. 21157483 10.1038/nrm3025 PMC3390257

